# Racial/ethnic and socioeconomic disparities in COVID-19 infections among working-age women with precancerous cervical lesion in Louisiana: analysis of more than two years of COVID-19 data

**DOI:** 10.3389/fepid.2023.1108452

**Published:** 2023-05-05

**Authors:** Mei-Chin Hsieh, Christina Lefante, Susanne Straif-Bourgeois, Yong Yi, Natalie Gomez, Pratibha Shrestha, Vivien W. Chen, Xiao-Cheng Wu

**Affiliations:** ^1^ Louisiana Tumor Registry, School of Public Health, Louisiana State University Health Sciences Center, New Orleans, LA, United States; ^2^ Epidemiology Program, School of Public Health, Louisiana State University Health Sciences Center, New Orleans, LA, United States

**Keywords:** COVID-19, racial/ethnic, SES, precancerous cervical lesion, women

## Abstract

**Background:**

Precancerous cervical lesion (PCL) is common in working-age and minority women. In Louisiana, 98% of PCL cases were diagnosed at age 18–65 with over 90% of them being human papillomavirus (HPV)-related. PCL women represent those who may be immunocompromised from the precancerous condition and thus more vulnerable to SARS-CoV-2. Most studies evaluating racial disparities for COVID-19 infection have only used data prior to vaccine availability. This study assessed disparities by race/ethnicity and socioeconomic status (SES) in COVID-19 infections among working-age PCL women for pre- and post-COVID-19 vaccine availability.

**Methods:**

Louisiana women aged 18–65 with PCL diagnosed in 2009–2021 were linked with the Louisiana statewide COVID-19 database to identify those with positive COVID-19 test. Race/ethnicity was categorized as non-Hispanic white (NHW), non-Hispanic black (NHB), Hispanic, and others. The census tract SES quintiles were created based on American Community Survey estimates. Logistic regression was employed to assess the racial/ethnic and SES differences in COVID-19 infections.

**Results:**

Of 14,669 eligible PCL women, 30% were tested COVID-19 positive. NHB had the highest percentage of COVID-19 infection (34.6%), followed by NHW (27.7%). The infection percentage was inversely proportional to SES, with 32.9% for women having the lowest SES and 26.8% for those with the highest SES. NHB women and those with lower SES had higher COVID-19 infection than their counterparts with an aOR of 1.37 (95% CI 1.25–1.49) and 1.21 (95% CI 1.07–1.37), respectively. In the pre-vaccine period, NHB and Hispanic women had higher odds of infection than NHW women. However, after the vaccine was implemented, the significant racial/ethnic and SES differences in COVID-19 infections still existed in PCL women residing in non-Greater New Orleans area.

**Conclusions:**

There are substantial variations in racial/ethnic and SES disparities in COVID-19 infections among working-age women with PCL, even after vaccine implementation. It is imperative to provide public health interventions and resources to reduce this unequal burden for this vulnerable population.

## Introduction

Severe acute respiratory syndrome coronavirus 2 (SARS-CoV-2), the causative agent of novel coronavirus disease-2019 (COVID-19), was first identified in late December 2019. This newly emerging virus rapidly spread worldwide and in 2020 COVID-19 was declared a pandemic. As of September 15, 2022, there were 95.4 million cumulative COVID-19 cases in the United States (U.S.) and 1.4 million in Louisiana (1). Data showed that COVID-19 Infection rates were highest among the young and middle-aged adults (2–6). Also, working age women had higher infection risk than men (3, 6).

Previous studies have shown that African Americans (AAs), Hispanic individuals, and geographic areas with higher percent population of color had a higher COVID-19 infection than their counterparts (7–15). Population density, socioeconomic status (SES), deprivation index, median income level, poverty, and diverse demographics are associated with the risk of COVID-19 infection (10, 11, 13–18). A hospitals-based study using data from Kaiser Foundation Hospitals showed AAs, Hispanic individuals, and Asians had increased likelihood of COVID-19 infection when compared with their white counterparts with an adjusted odds ratio (aOR) of 2.01 [95% confidence interval (CI) 1.75–2.31], 3.93 (95% CI 3.59–4.30), and 2.19 (95% CI 1.98–2.42), respectively (11). Studies also demonstrated that a high deprivation index correlated with an increased likelihood of being infected with COVID-19 (11, 16). However, most studies assessing racial and/or SES disparities in COVID-19 infection had have used data prior to COVID-19 vaccination implementation (7–18). As a result, it is unclear whether these disparities have persisted following the availability of the COVID-19 vaccine to the general population.

Although the racial disparity in COVID-19 infection among cancer patients was studied using electronic health records from 360 hospitals in the U.S. (7), women with precancerous cervical lesion (PCL) were not part of this study. According to the U.S. Cancer Statistics (USCS), 58% of cancers are found in adults aged 65 years or older (19); however, PCL is more likely to be diagnosed in younger, working aged women and women residing in census tract with higher levels of poverty and larger proportions of black residents had higher PCL incidence rates (20). In Louisiana, over 98% of PCL was diagnosed in women aged 18–65, with women aged 20–34 having the highest incidence rates (21). Studies found that SARS-CoV-2, the causative agent of COVID-19, could directly infect the cervical epithelium or dysregulate the immune system by preoccupying and exhausting the system resulting in cervical dysplasia or rapid progression of existing lesions (22–24). The main risk factor for acquiring PCL is human papillomavirus (HPV) infection. In the U.S., over 90% of cervical neoplasia are HPV-related (25, 26). Therefore, women with PCL could be more susceptible to SARS-CoV-2 due to their immunocompromised status and there were no studies assessing their risk.

The COVID-19 pandemic also impacted the Louisiana PCL incidence between 2019 and 2020, which declined about 15%. This is mainly attributed to missed routine cervical screening due to state lock-down in the first 3 months of pandemic and hesitation to interact with the healthcare community (27). The overall cervical screen rate decreased about 9% in 2020, especially for white and aged 21–24 women decreased about 13% and 15%, respectively (28). Louisiana Tumor Registry (LTR), a statewide population-based cancer registry, is one of only four U.S. population-based cancer registries initially funded by the Centers for Disease Control and Prevention (CDC) and subsequently funded by the state to collect incidence of PCL for assessing the population-level impact of HPV vaccination; thus, providing a unique opportunity to study this subgroup. This study aims to evaluate the racial/ethnic disparities and to examine the socioeconomic variations in COVID-19 infections among working-age women with PCL in Louisiana including pre- and post-COVID-19 vaccine availability.

## Methods and materials

### Data source and study population

**Figure 1:**
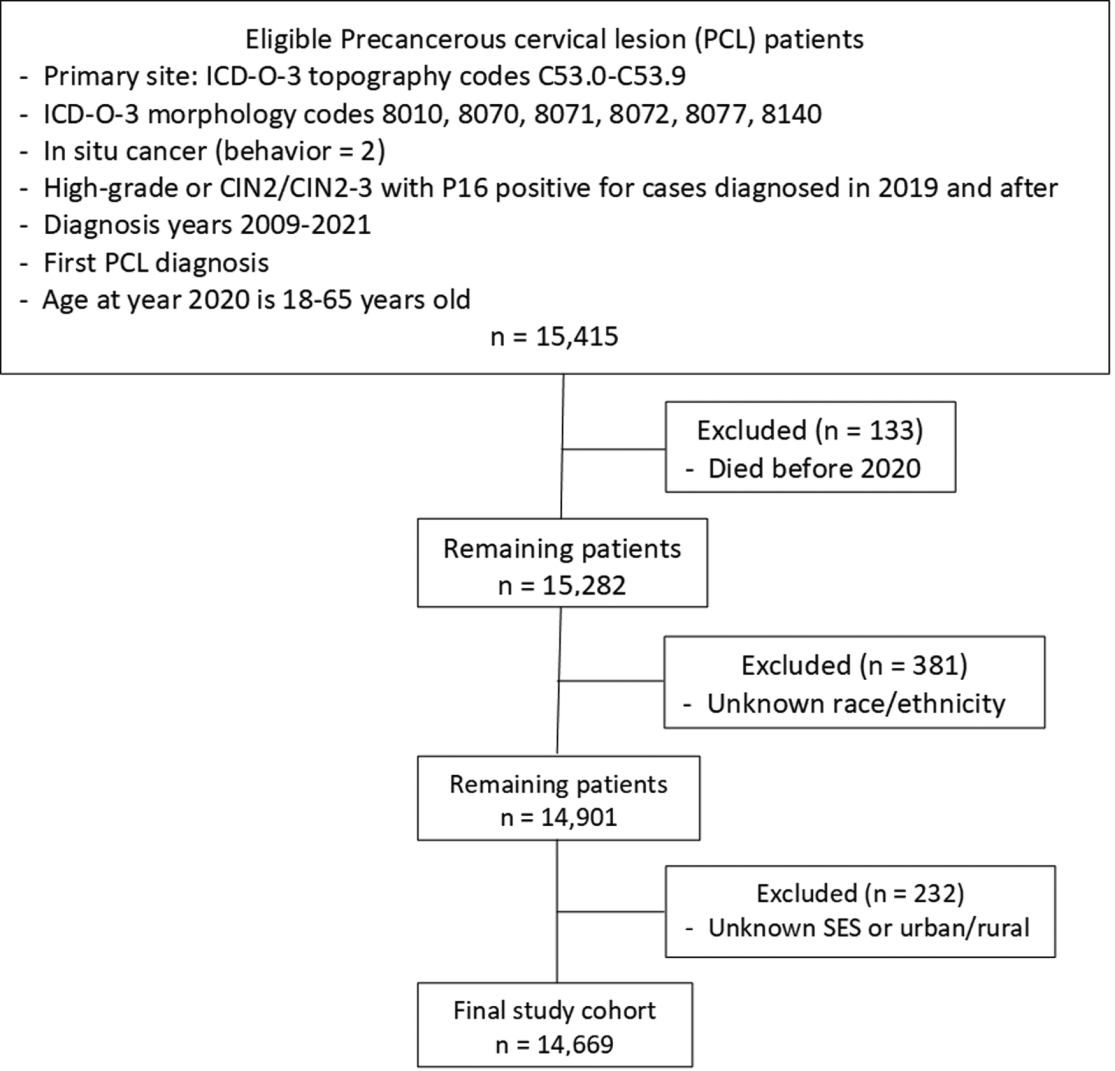
Flowchart of study cohort selection. ICD-O-3, international classification of disease for oncology, 3rd edition; CIN2, cervical intraepithelial neoplasia, grade 2; SES, socioeconomic status.

Women with PCL diagnosed in 2009–2021 were obtained from the LTR. The collection criteria of PCL included cervical intraepithelial neoplasia grade III (CIN3), carcinoma *in situ* (CIS), severe dysplasia, adenocarcinoma *in situ* (AIS), and high-grade dysplasia or CIN2/CIN2–3 with positive P16 test in diagnosis year 2019 and afterward. We defined working-age PCL women as women aged 18–65 in 2020. PCL women who died before 2020, with unknown race/ethnicity or missing SES were excluded. The detailed inclusion and exclusion criteria are illustrated in Figure 1.

### COVID-19 data

The Louisiana statewide COVID-19 database was established in March 2020 by the Louisiana Department of Health (LDH) to collect COVID-19 test data. COVID-19 database is managed and maintained by the Infectious Disease Epidemiology Section at the Louisiana Office of Public Health in New Orleans (29). This database contains patient-level data on demographics, first and last date of COVID-19 antigen and/or PCR test, and test results. The COVID-19 test data was mainly reported by clinical setting, pharmacies, and stand-alone testing sites. All eligible PCL women were linked with the statewide COVID-19 data from March 2020 to March 2022, including pre-vaccine and post-vaccine period, to identify patients with a positive COVID-19 test. For individual with multiple COVID-19 positive dates, only the date of first COVID-19 test positive or date of first PCR test positive was selected.

### Variables

The outcome variable of this study is COVID-19 infection (Yes vs. No). The COVID-19 infection was defined based on the first positive test result either through antigen or PCR test. The explanatory variables of interest were race/ethnicity and socioeconomic status (SES). We categorized race/ethnicity into non-Hispanic white (NHW), non-Hispanic black (NHB), Hispanic, and non-Hispanic others which includes American Indian/Alaska Native, Asian, and Pacific Islander. We applied the North American Association of Central Cancer Registries (NAACCR) Hispanic/Latino Identification Algorithm (NHIA v2.2) to determine the Hispanic ethnicity (30). There are five race fields (Race 1–Race 5) in the registries database to allow the coding of multiple races for a person and a separate Spanish/Hispanic origin code. The Surveillance, Epidemiology and End Results (SEER) Program standard race coding guidelines and priorities for coding multiple races was used to code race(s) (31). Only Race 1 was used to categorize race group in this study. Hispanic individuals were those with NHIA coded to 1 (Hispanic ethnicity) regardless the race. Due to a very small number of PCL women having race as American Indian/Alaska Native, Asian, and Pacific Islander in LTR database, they were grouped as non-Hispanic others. The SES quintiles (higher group indicates the higher SES) were created using seven census tract-level SES attributes, which included median household income, median house value, median rent, percent below 150% of poverty line, education index, percent working class, and percent unemployed obtained from the American Community Survey (ACS) 5-year estimates. Census tracts encompassed areas that are more homogeneous with respect to population characteristics than county or zip code level SES (32).

We characterized geographic location as Greater New Orleans (GNO) area and non-GNO area. The GNO area, which includes four parishes (Orleans, Jefferson, Saint Bernard, and Plaquemines) located in southeast Louisiana, has historically had a higher proportion of non-white population than the rest of state. The non-GNO area included the remaining 60 parishes. Other covariates included age at year 2020 (aged 18–29, 30–39, 40–49, 50–65), marital status (married including living with domestic partner, single including never married/separated/divorced, and unknown), type of health insurance (private, Medicare/other public, Medicaid, and uninsured/unknown), PCL histology type (AIS vs. non-AIS), and urban-rural status. We used Urban/Rural Indicator Code (URIC) to define urban-rural at the census tract level. The URIC is based on the Census Bureau's percent of the population living in non-urban areas, which were categorized as 100% urban (All urban), ≥50% to <100% urban (Mostly urban), >0% to <50% urban (Mostly rural), and 100% rural (All rural) tracts. We used December 31, 2020 as the cutoff date to define pre- and post-COVID-19 vaccine period.

### Statistical analysis

Descriptive statistics on covariates by race/ethnicity were presented and the chi-square test was used to measure the unadjusted association. The logistic regression models with the Firth correction to control the rare events bias was employed to assess the unadjusted and adjusted effect of racial/ethnic and SES differences in COVID-19 infections among working-age women with PCL disease in Louisiana. The unadjusted odds ratio (OR) and adjusted OR (aOR) for each covariate with the corresponding 95% confidence interval (CI) were presented. We further examined the disparities of race/ethnicity and SES in COVID-19 test positive cases stratified by age group as well as geographic location to examine the variations. An additional data analysis was conducted to evaluate the disparities of race/ethnicity and SES in COVID-19 infections prior to and after a COVID-19 vaccine being widely available to general population in the U.S., which covered the period of March 2020 and December 2020 and the period of January 2021 and March 2022. SAS version 9.4 (SAS Institute, Inc., Cary, NC) was used to carry out all data analyses and tests were performed at significance levels of 0.05.

## Results

**Figure 2:**
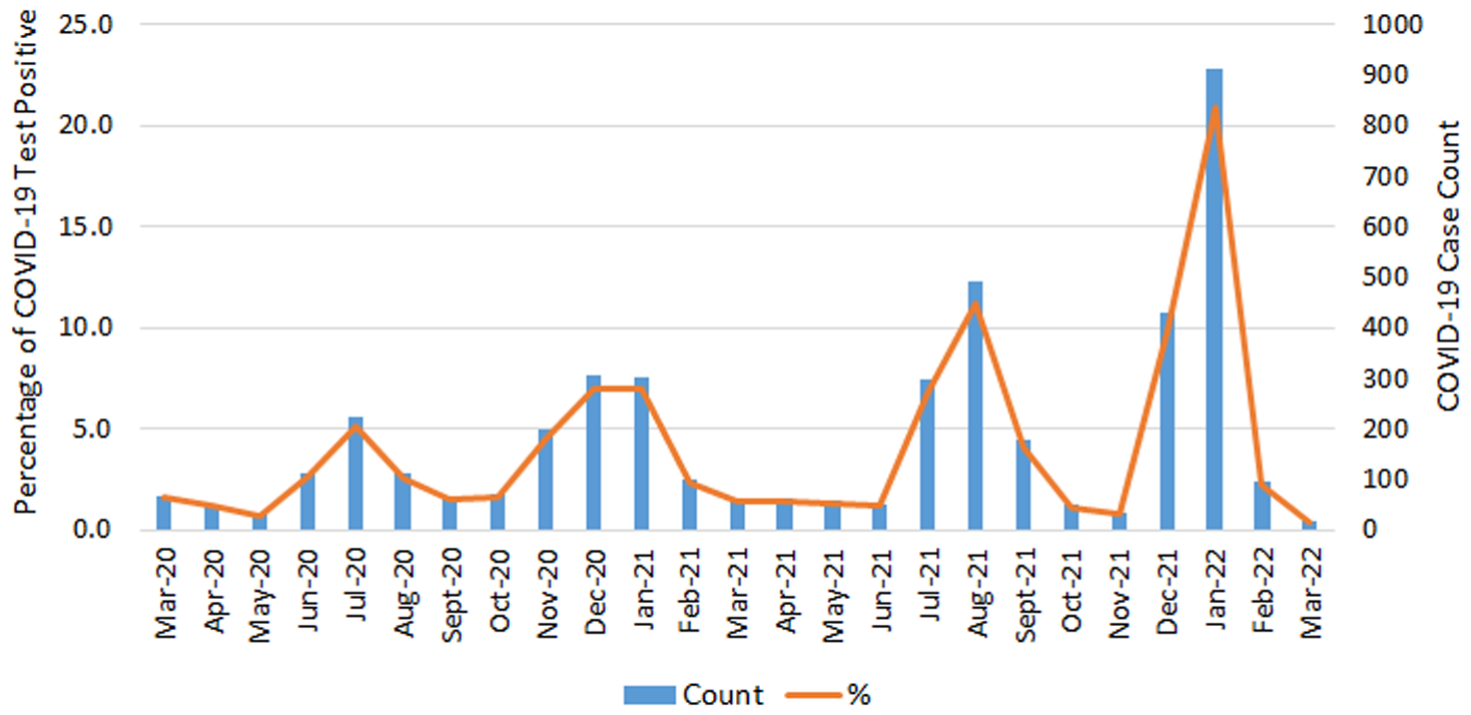
COVID-19 positive case count and percentage by month of testing positive among PCL women in Louisiana. PCL, precancerous cervical lesion.

Among 14,669 eligible PCL women, 60.4% were NHW, 32.7% were NHB, 5.4% were Hispanic, and only 1.5% were other races/ethnicities. About 30% (4,368) of PCL women were tested with COVID-19 positive (Table 1) and three quarters of them were diagnosed through a positive PCR test. NHB had the highest percentage of COVID-19 infections (34.6%), followed by NHW (27.7%) (Table 1). Hispanic women and other race/ethnicity had a similar lower percentage of infections at 25.5% and 25.1%, respectively. NHW and other race/ethnicity PCL women were more likely residing in the higher SES areas (Table 1). Figure 2 shows the frequency distribution of COVID-19 test positive cases by month among PCL women in Louisiana. The peaks were noted in summer and winter. Around 20.9% of COVID-19 cases were found in January 2022 followed by August 2021 (11.3%). The percentage of COVID-19 infections decreased as age increased, with 36.8% for PCL women aged 18–29 and 23.6% for those aged 50–65. A similar pattern appeared in SES with infection percentage being higher (32.9%) for women residing in the lowest SES census tracts and declining (26.8%) for those residing in the highest SES areas (Table 2).

**Table 1:** Characteristics among working-age women with PCL diagnosed in 2009–2021 by race/ethnicity in Louisiana.

Variables	Race/Ethnicity				Total, *N* (%)	*p*-value
	NHW *N* (%)	NHB *N* (%)	Hispanic *N* (%)	Other^a^ *N* (%)		
COVID-19 infection						<.0001
No	6,404 (72.3)	3,138 (65.4)	595 (74.5)	164 (74.9)	10,301 (70.2)	
Yes	2,451 (27.7)	1,658 (34.6)	204 (25.5)	55 (25.1)	4,368 (29.8)	
Age						<.0001
18–29	1,370 (15.5)	811 (16.9)	143 (17.9)	21 (9.6)	2,345 (16.0)	
30–39	4,375 (49.4)	2,385 (49.7)	391 (48.9)	101 (46.1)	7,252 (49.4)	
40–49	2,083 (23.5)	1,010 (21.1)	196 (24.5)	64 (29.2)	3,353 (22.9)	
50–65	1,027 (11.6)	590 (12.3)	69 (8.6)	33 (15.1)	1,719 (11.7)	
Marital status						<.0001
Married	825 (9.3)	248 (5.2)	77 (9.6)	23 (10.5)	1,173 (8.0)	
Single	1,206 (13.6)	1,056 (22.0)	204 (25.5)	25 (11.4)	2,491 (17.0)	
Unknown	6,824 (77.1)	3,492 (72.8)	518 (64.8)	171 (78.1)	11,005 (75.0)	
Health insurance						<.0001
Private/Medicare/Other public	788 (8.9)	285 (5.9)	44 (5.5)	17 (7.8)	1,134 (7.7)	
Medicaid	337 (3.8)	342 (7.1)	28 (3.5)	16 (7.3)	723 (4.9)	
No insurance/unknown	7,730 (87.3)	4,169 (86.9)	727 (91.0)	185 (84.9)	12,812 (87.3)	
SES						<.0001
Group 1 (Lowest)	842 (9.5)	2,015 (42.0)	125 (15.6)	26 (11.9)	3,008 (20.5)	
Group 2	1,631 (18.4)	1,054 (22.0)	189 (23.7)	41 (18.7)	2,915 (19.9)	
Group 3	1,965 (22.2)	713 (14.9)	147 (18.4)	30 (13.7)	2,855 (19.5)	
Group 4	2,237 (25.3)	585 (12.2)	169 (21.2)	59 (26.9)	3,050 (20.8)	
Group 5 (Highest)	2,180 (24.6)	429 (8.9)	169 (21.2)	63 (28.8)	2,841 (19.4)	
Urban/Rural						<.0001
All urban (100% urban)	2,930 (33.1)	2,807 (58.5)	519 (65.0)	120 (54.8)	6,376 (43.5)	
Mostly urban (≥50% to <100% urban)	3,164 (35.7)	1,414 (29.5)	203 (25.4)	69 (31.5)	4,850 (33.1)	
Mostly rural (>0% to <50% urban)	1,648 (18.6)	321 (6.7)	54 (6.8)	17 (7.8)	2,040 (13.9)	
All rural (100% rural)	1,113 (12.6)	254 (5.3)	23 (2.9)	13 (5.9)	1,403 (9.6)	
Geographic location						<.0001
GNO	1,032 (11.7)	1,016 (21.2)	351 (43.9)	73 (33.3)	2,472 (16.9)	
Non-GNO	7,823 (88.4)	3,780 (78.8)	448 (56.1)	146 (66.7)	12,197 (83.1)	
Histology type						<.0001
Non-AIS	8,625 (97.4)	4,766 (99.4)	783 (98.0)	213 (97.3)	14,387 (98.1)	
AIS	230 (2.6)	30 (0.6)	16 (2.0)	6 (2.7)	282 (1.9)	

PCL, precancerous cervical lesion; NHW, non-hispanic white; NHB, non-hispanic black; SES, socioeconomic status; GNO, Greater New Orleans; AIS, adenocarcinoma *in situ*.

^a^ Other race/ethnicity includes American Indian/Alaska Native, Asian, and pacific islanders.

**Table 2:** Characteristics of women with PCL by COVID-19 infection and the association of factors in COVID-19 infection among working-age PCL women, Louisiana.

Variables	COVID-19 infection		Unadjusted OR (95% CI)	Adjusted OR (95% CI)
	No, *N* (%)	Yes, *N* (%)		
Race/Ethnicity				
NHW	6,404 (72.3)	2,451 (27.7)	1.00	1.00
NHB	3,138 (65.4)	1,658 (34.6)	1.38 (1.28–1.49)	1.37 (1.25–1.49)
Hispanic	595 (74.5)	204 (25.5)	0.90 (0.76–1.06)	0.91 (0.77–1.08)
Other^a^	164 (74.9)	55 (25.1)	0.88 (0.65–1.20)	0.95 (0.70–1.29)
Age				
18–29	1,482 (63.2)	863 (36.8)	1.89 (1.64–2.17)	1.90 (1.65–2.18)
30–39	5,115 (70.5)	2,137 (29.5)	1.35 (1.20–1.53)	1.37 (1.21–1.54)
40–49	2,390 (71.3)	963 (28.7)	1.31 (1.14–1.49)	1.33 (1.16–1.52)
50–65	1,314 (76.4)	405 (23.6)	1.00	1.00
Marital status				
Married	808 (68.9)	365 (31.1)	1.00	1.00
Single	1,708 (68.6)	783 (31.4)	1.01 (0.87–1.18)	0.87 (0.75–1.02)
Unknown	7,785 (70.7)	3,220 (29.3)	0.92 (0.80–1.04)	0.81 (0.71–0.94)
Health insurance				
Private/Medicare/Other public	788 (69.5)	346 (30.5)	1.00	1.00
Medicaid	512 (70.8)	211 (29.2)	0.94 (0.77–1.15)	0.84 (0.68–1.03)
No insurance/unknown	9,001 (70.3)	3,811 (29.8)	0.96 (0.84–1.10)	0.93 (0.81–1.08)
SES				
Group 1 (Lowest)	2,020 (67.2)	988 (32.9)	1.34 (1.19–1.50)	1.21 (1.07–1.37)
Group 2	2,028 (69.6)	887 (30.4)	1.20 (1.07–1.34)	1.16 (1.03–1.30)
Group 3	2,013 (70.5)	842 (29.5)	1.14 (1.02–1.28)	1.12 (0.99–1.27)
Group 4	2,160 (70.8)	890 (29.2)	1.13 (1.01–1.26)	1.11 (0.99–1.25)
Group 5 (Highest)	2,080 (73.2)	761 (26.8)	1.00	1.00
Urban/Rural				
All urban (100% urban)	4,517 (70.8)	1,859 (29.2)	1.00	1.00
Mostly urban (≥50% to <100% urban)	3,362 (69.3)	1,488 (30.7)	1.08 (0.99–1.17)	1.13 (1.03–1.23)
Mostly rural (>0% to <50% urban)	1,413 (69.3)	627 (30.7)	1.08 (0.97–1.20)	1.18 (1.05–1.34)
All rural (100% rural)	1,009 (71.9)	394 (28.1)	0.95 (0.84–1.08)	1.02 (0.88–1.17)
Geographic location				
GNO	1,768 (71.5)	704 (28.5)	1.00	1.00
Non-GNO	8,533 (70.0)	3,664 (30.0)	1.08 (0.98–1.19)	1.04 (0.94–1.17)
Histology type				
Non-AIS	10,077 (70.0)	4,310 (30.0)	1.64 (1.23–2.19)	1.46 (1.08–1.95)
AIS	224 (79.4)	58 (20.6)	1.00	1.00

PCL, precancerous cervical lesion; OR, odds ratio; NHW, non-hispanic white; NHB, non-hispanic black; SES, socioeconomic status; GNO, Greater New Orleans; AIS, adenocarcinoma *in situ*.

^a^ Other race/ethnicity includes American Indian/Alaska Native, Asian, and pacific islanders.

**Figure 3:**
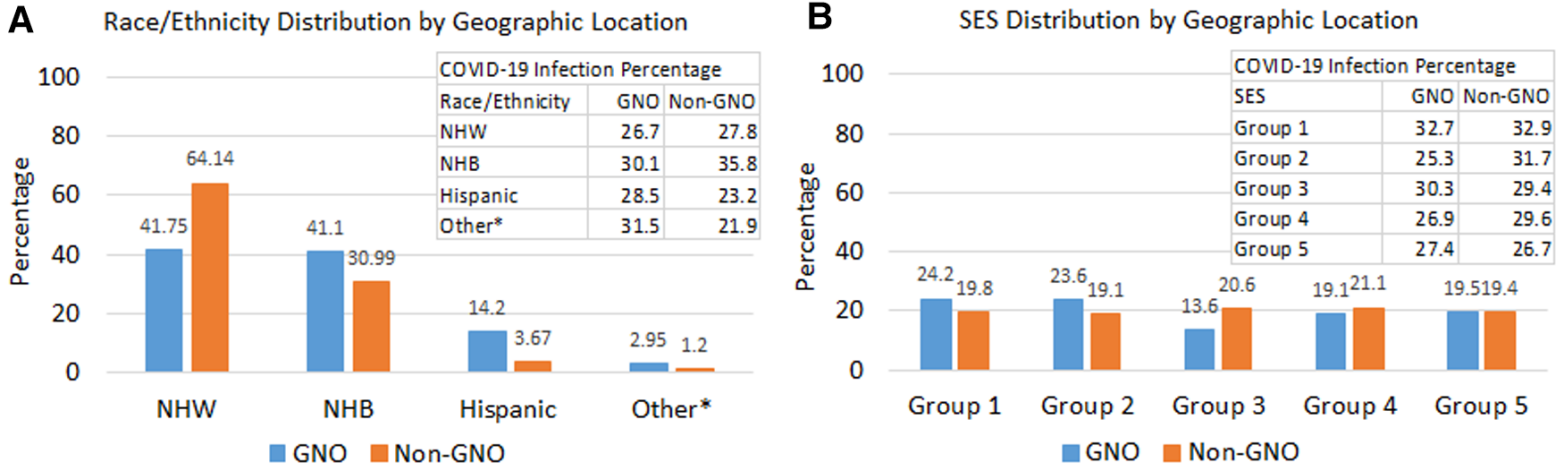
Distributions of race/ethnicity, SES, and COVID-19 infection by geographic location. (**A**) Race/ethnicity distribution by geographic location and COVID-19 infection percentage by race/ethnicity and geographic location. (**B**) SES distribution by geographic location and COVID-19 infection percentage by SES and geographic location. *Other race/ethnicity includes American Indian/Alaska Native, Asian, and pacific islanders. PCL, precancerous cervical lesion; NHW, non-hispanic white; NHB, non-hispanic black; SES, socioeconomic status; GNO, Greater New Orleans.

The distributions of race/ethnicity and SES were relatively different between GNO and non-GNO PCL women (Figure 3). The proportion of NHW and NHB in the GNO area were similar, 41.8% vs. 41.1%; whereas, NHW PCL women (64.1%) in the non-GNO were double the size of NHB women (31.0%) (Figure 3A). While, NHW had a comparable COVID-19 infection percentage between GNO and non-GNO, 26.7% and 27.8%, respectively; NHB PCL women residing in the non-GNO area had a much higher proportion of COVID-19 infection (35.8%) than those in the GNO area (30.1%). The distribution of SES in the non-GNO area was fairly even across quintiles with percentage of COVID-19 infection decreased as SES increased; but this was not observed in the GNO area with about 48% PCL women living in lower SES census tracts (Figure 3B).

**Figure 4:**
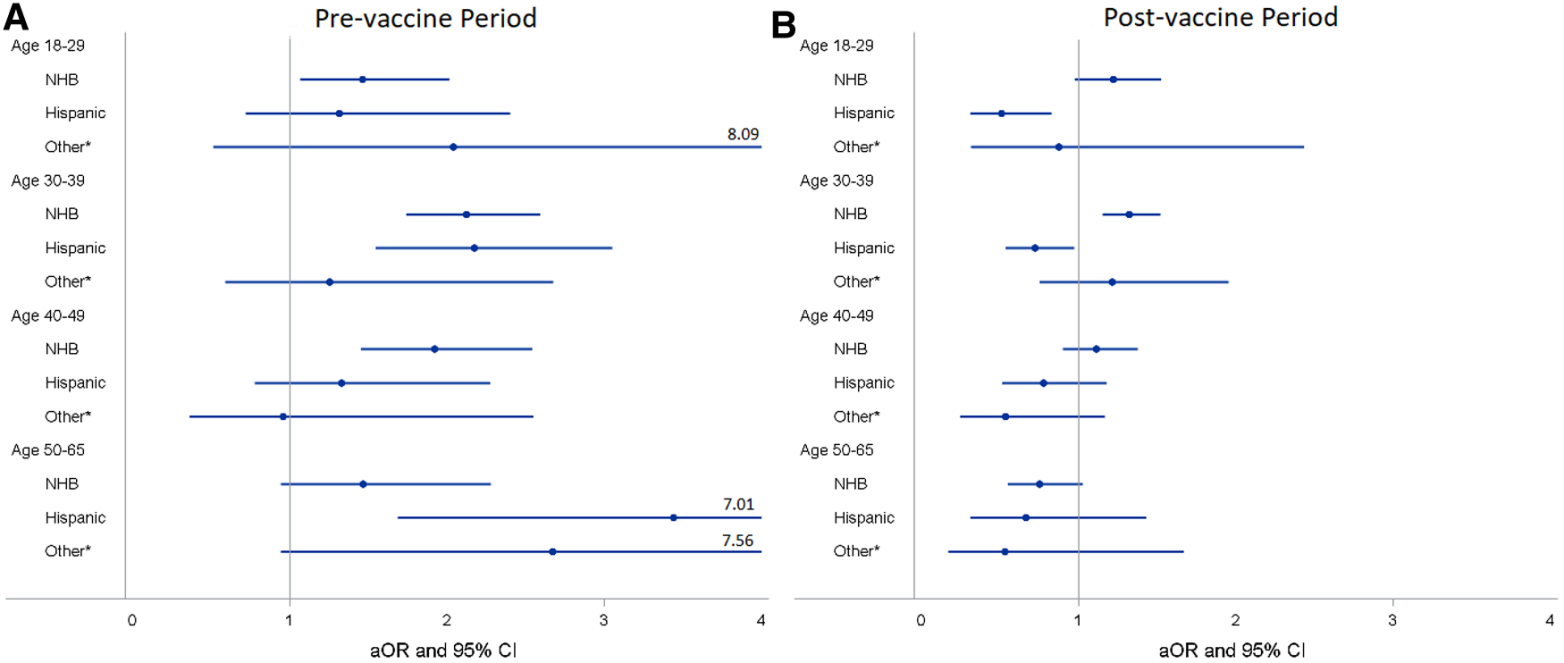
Adjusted odds ratios^a^ (aORs) and 95% confidence intervals (CIs) for race/ethnicity stratified by vaccine period and age group for PCL women in Louisiana. (**A**) aOR^a^ and 95% CI of racial/ethnic groups stratified by age group for pre-vaccine period. (**B**) aOR^a^ and 95% CI of racial/ethnic groups stratified by age group for post-vaccine period. ^a^Adjusted for marital status, health insurance, socioeconomic status, urban/rural, geographic location, and histology type. *Other race/ethnicity includes American Indian/Alaska Native, Asian, and pacific islanders. PCL, precancerous cervical lesion; NHW, non-hispanic white; NHB, non-hispanic black.

We found that NHB PCL women had higher odds of COVID-19 infection than NHW with an unadjusted OR of 1.38 (95% CI 1.28–1.49) and an aOR of 1.37 (95% CI 1.25–1.49) (Table 2). Compared to women aged 50–65, younger age groups were more likely to have COVID-19 test positive with an aOR of 1.90 (95% CI 1.65–2.18) for those aged 18–29, 1.37 (95% CI 1.21–1.54) for aged 30–39, and 1.33 (95% CI 1.16–1.52) for aged 40–49. After stratifying by vaccine period and age group, NHB women had a higher probability of COVID-19 infection compared to NHW women across all age groups except for women of age 50–65 before vaccine available to the general population, with an aOR of 1.46 (95% CI 1.07–2.01) for aged 18–29, 2.12 (95% CI 1.74–2.59) for aged 30–39, and 1.92 (95% CI 1.45–2.54) for aged 40–49 (Figure 4A). However, after vaccine was implemented, only NHB women aged 30–39 remained a higher likelihood of infection than NHW women with an aOR decreasing to 1.32 (95% CI 1.15–1.52) (Figure 4B). For Hispanic PCL women, we found women aged 30–39 and 50–65 were more likely to have tested positive for COVID-19 than NHW during the pre-vaccine period with an aOR of 2.17 (95% CI 1.55–3.05) and 3.44 (1.69–7.01), respectively (Figure 4A). Conversely, Hispanic women aged 18–29 and 30–39 had much lower odds of COVID-19 infection than NHW with an aOR of 0.51 (95% CI 0.31–0.83) and 0.72 (95% CI 0.54–0.97) after COVID-19 vaccine was widely available to the general population (Figure 4B).

SES and urban/rural status were also significantly associated with COVID-19 infection among PCL women (Table 2). Women residing in the lower SES census tracts had increased odds for COVID-19 infection when compared to those residing in the highest SES (Group 5), with an aOR of 1.21 (95% CI 1.07–1.37) for SES group 1 and 1.16 (95% CI 1.03–1.31) for SES group 2 (Table 2). When compared to PCL women residing in 100% urban (all urban), women residing in mixed urban/rural area had a higher likelihood of COVID-19 infection with an aOR of 1.13 (95% CI 1.03–1.23) for mostly urban area and 1.18 (95% CI 1.05–1.34) for mostly rural area.

Table 3 shows the aORs and 95% CIs of race/ethnic and SES in COVID-19 infection stratified by geographic location. We did not observe a significant association in COVID-19 infection, neither for race/ethnicity nor for SES among PCL women residing in the GNO area after controlling for age, marital status, health insurance, urban/rural, and histology type. Contrarywise, for PCL women residing in the non-GNO area, NHB had 42% (aOR 1.41, 95% CI 1.29–1.56) higher odds and Hispanic had 23% (aOR 0.77, 95% CI 0.61–0.97) lower odds of COVID-19 infection than NHW women. Women with SES in group 1, group 2, and group 4 were more likely to have COVID-19 diagnosis compared to those in group 5, with an aOR of 1.20 (95% CI 1.05–1.38), 1.24 (95% CI 1.08–1.41), and 1.15 (95% CI 1.01–1.30), respectively.

**Table 3:** Adjusted odds ratios^a^ and 95% confidence intervals of race/ethnicity and SES in COVID-19 infection among working-age PCL women by geographic location, Louisiana.

Variables	GNO (*N* = 2,472)	Non-GNO (*N* = 12,197)
Race/Ethnicity		
NHW	1.00	1.00
NHB	1.17 (0.94–1.45)	1.42 (1.29–1.56)
Hispanic	1.11 (0.84–1.46)	0.77 (0.61–0.97)
Other^b^	1.32 (0.79–2.21)	0.78 (0.52–1.16)
SES		
Group 1 (Lowest)	1.21 (0.90–1.62)	1.20 (1.05–1.38)
Group 2	0.86 (0.65–1.15)	1.24 (1.08–1.41)
Group 3	1.10 (0.80–1.51)	1.13 (0.99–1.29)
Group 4	0.94 (0.71–1.26)	1.15 (1.01–1.30)
Group 5 (Highest)	1.00	1.00

SES, socioeconomic status; PCL, precancerous cervical lesion; NHW, non-hispanic white; NHB, non-hispanic black; GNO, Greater New Orleans.

^a^ Adjusted for age, marital status, health insurance, urban/rural, and histology type.

^b^ Other race/ethnicity includes American Indian/Alaska Native, Asian, and pacific islanders.

The aORs and 95% CIs of race/ethnicity and SES in COVID-19 infections stratified by geographic location for pre- and post-COVID-19 vaccine period were presented in Table 4. We found that NHB women had a higher likelihood of COVID-19 infection than NHW women for both pre- and post-vaccine period in Louisiana as a whole, with an aOR of 1.89 (95% CI 1.64–2.17) and 1.18 (95% CI 1.07–1.41), respectively. Compared to NHW, Hispanic women had higher odds of COVID-19 infection in pre-vaccine and lower odds in post-vaccine, aOR 1.83 (95% CI 1.43–2.33) and aOR 0.67 (0.54–0.83), respectively. After stratified by geographic area, NHB women residing in either the GNO (aOR 2.13, 95% CI 1.81–2.49) or non-GNO (aOR 1.76, 95% CI 1.17–2.64) area had higher odds of infection as compared to NHW women during the pre-vaccine period. However, in post-vaccine period, the significant correlation only observed for NHB women residing in the non-GNO (aOR 1.22, 95% CI 1.10–1.36) area (Table 4). Hispanic women also had higher odds of infection compared to NHW women in both GNO (aOR 2.53, 95% CI 1.67–3.83) and non-GNO (aOR 1.41, 95% CI 1.01–1.96) areas in the pre-vaccine period; yet, the corrections altered to different directions after vaccine was implemented with an aOR of 0.75 (95% CI 0.54–1.05) for GNO and 0.61 (95% CI 0.46–0.81) for non-GNO. Furthermore, the association for other race/ethnicity altered from significantly higher than NHW (aOR 2.17, 95% CI 1.01–4.68) before vaccine availability to not statistically significant after vaccine implementation among women in GNO area. Based on pre-vaccine data, we only found PCL women residing in group 4 had higher risk of COVID-19 infection than those in group 5 in the GNO area (aOR 1.83, 95% CI 1.13–2.95) (Table 4). In contract, PCL women residing in the non-GNO area with lower SES (groups 1 and 2) had a significantly higher likelihood of infection than those with the highest SES (group5) in post-vaccine period, with an aOR of 1.25 (1.07–1.46) and aOR 1.32 (1.14–1.53), respectively.

**Table 4:** Adjusted odds ratios^a^ and 95% confidence intervals of race/ethnicity and SES on COVID-19 infection among PCL women^b^ by geographic location in Louisiana for pre- and post-COVID-19 vaccine period^c^.

Variables	Louisiana	GNO	Non-GNO
Pre-vaccine period (3/1/2020–12/31/2020)			
COVID-19 positive	1,237 (8.4%)	200 (8.1%)	1,037 (8.5%)
Race/Ethnicity			
NHW	1.00	1.00	1.00
NHB	1.89 (1.64–2.17)	1.64 (1.13–2.38)	1.94 (1.68–2.25)
Hispanic	1.83 (1.43–2.33)	2.53 (1.67–3.83)	1.41 (1.01–1.96)
Other^d^	1.28 (0.78–2.10)	2.17 (1.01–4.68)	0.98 (0.51–1.91)
SES			
Group 1 (Lowest)	1.06 (0.87–1.30)	1.19 (0.71–2.00)	1.03 (0.82–1.29)
Group 2	1.00 (0.81–1.22)	1.05 (0.63–1.75)	0.98 (0.78–1.22)
Group 3	1.10 (0.90–1.35)	1.40 (0.82–2.40)	1.04 (0.83–1.30)
Group 4	1.29 (1.07–1.57)	1.83 (1.13–2.95)	1.20 (0.97–1.48)
Group 5 (Highest)	1.00	1.00	1.00
Post-vaccine period (1/1/2021–3/31/2022)			
COVID-19 positive	3,131 (23.3%)	504 (22.2%)	2,627 (23.5%)
Race/Ethnicity			
NHW	1.00	1.00	1.00
NHB	1.18 (1.07–1.31)	1.02 (0.80–1.31)	1.22 (1.10–1.36)
Hispanic	0.67 (0.54–0.83)	0.75 (0.54–1.05)	0.61 (0.46–0.81)
Other^d^	0.86 (0.60–1.23)	1.12 (0.61–2.03)	0.74 (0.47–1.16)
SES			
Group 1 (Lowest)	1.24 (1.08–1.43)	1.22 (0.88–1.69)	1.25 (1.07–1.46)
Group 2	1.21 (1.05–1.38)	0.84 (0.61–1.15)	1.32 (1.14–1.53)
Group 3	1.12 (0.98–1.29)	1.02 (0.71–1.45)	1.15 (0.99–1.34)
Group 4	1.04 (0.91–1.19)	0.73 (0.52–1.02)	1.12 (0.97–1.29)
Group 5 (Highest)	1.00	1.00	1.00

SES, socioeconomic status; PCL, precancerous cervical lesion; NHW, non-hispanic white; NHB, non-hispanic black; GNO, Greater New Orleans.

^a^ Adjusted for age, marital status, health insurance urban/rural, and histology type for GNO and non-GNO and added geographic location to the adjusted model for Louisiana as a whole.

^b^ Included PCL cases diagnosed between 2009 and 2020.

^c^ Used December 31, 2020 as the cutoff date for pre- and post-COVID-19 vaccine period.

^d^ Other race/ethnicity includes American Indian/Alaska Native, Asian, and pacific islanders.

## Discussion

In this retrospective population-based study of women with PCL, the data revealed substantial racial/ethnic and SES disparities in COVID-19 infections. We observed that NHB and women residing in lower SES census tract had an increased likelihood of COVID-19 infection compared to their counterparts. After COVID-19 vaccine being available to the general population, the racial/ethnic and SES disparities in COVID-19 infection were persistently existed among PCL women who resided outside of the Greater New Orleans (GNO) area.

Since 1996, PCL cases were no longer required to be collected and reported to cancer surveillance organizations; only a few central cancer registries in the U.S. continued to collect PCL cases with state support and/or CDC funds. Therefore, to our knowledge, this is the first population-based study that evaluated the racial/ethnic and socioeconomic disparities in COVID-19 infection among this unique population.

The findings of racial/ethnic disparities in COVID-19 infections among working-age PCL women from this study varied with the results from previous studies except for the higher risk of COVID-19 infection among NHB than NHW which was consistently observed in both pre- and post-vaccine periods (7–15). Predominantly, NHB PCL women residing in the non-GNO area have much higher odds of COVID-19 infection than all other race/ethnicity groups. While some studies reported that Hispanic individuals had a higher risk of COVID-19 infection than their white counterparts (11–13), our results based on pre-vaccine data were consistent with previous studies (7, 8, 11–13). However, after included post-vaccine data, we did not find significant difference between NHW and Hispanic PCL women regarding COVID-19 infection in Louisiana as a whole. Conversely, after being stratified by geographic location, Hispanic PCL women who resided in the non-GNO area were, in fact, less likely to have a COVID-19 diagnosis than NHW women, which was in contrast with prior studies, particularly in the post-vaccine period. The decrease in racial/ethnic disparities in COVID-19 infections among PCL women could be attributed to COVID-19 vaccine uptake. Data on COVID-19 vaccinations showed 69% of Hispanic residents and 64% of black residents received at least one dose of vaccine in Louisiana compared to only 57% of whites as of July 14, 2022 (33).

The effect of socioeconomic indicators in COVID-19 infections was diverse in prior studies conducted in the U.S. A study found counties with higher education and income level were at a higher risk of COVID-19 infection (12); however, Chen et al. study, based on the zip code level poverty, showed that the COVID-19 incidence rate increased as percent poverty increased (10). Also, high deprivation index was associated with increase of COVID-19 infection (9, 11, 16). A study used publicly available aggregate COVID-19 data obtained from the Louisiana Department of Health website between March 2020 and July 2020 found individuals residing in the most deprived neighborhoods had a 39% (Risk ratio 1.39, 95% CI 1.27–1.52) higher risk of COVID-19 infection compared to those residing in the least deprived neighborhoods (16). Our data also showed that the probability of COVID-19 infection was directly correlated with SES. Louisiana PCL women residing in the lowest SES (the least affluent) census tract have the highest odds of COVID-19 infection and the odds of infection declines as SES increases which is consistent with previous studies that used the deprivation index (9, 11, 16). Similarly to the race/ethnicity, the SES disparities in Louisiana existed in PCL women residing in the non-GNO area after vaccine was implemented.

The different findings of disparities between GNO and non-GNO area, based on 2 years of COVID-19 data, could be primarily explained by differences in COVID-19 vaccination rates. Approximately 75.5% of population in the GNO areas received at least one dose of vaccine and 67.5% were fully vaccinated, which were much higher than those in the non-GNO areas, 54.3% received at least one dose and only 49.0% fully vaccinated (34). High vaccination uptake likely decreased the likelihood for the chain of events leading to spreading infections, therefore, the high vaccination rate in the GNO area may have contributed to the diminishing racial/ethnic and SES disparities among PCL women in this area. Underlying health condition is also a stronger predictor of COVID-19 incidence in Louisiana, particularly for NHB population. Kodsup's study found that the high-risk areas for NHB COVID-19 incidence rates are associated with parishes that had diabetes and obesity higher than 75th percentile in Louisiana (14). All these high-risk areas are located outside of the GNO area, this could partially explain why NHB had higher odds of COVID-19 infection than NHW among PCL women residing in the non-GNO area. Other reasons that caused racial/ethnic and SES disparities in COVID-19 infections among PCL women could include NHB women or women residing in lower SES area may have jobs that required them to work on site and/or living in crowded housing making it difficult to maintain social distancing increasing the risk of COVID-19 infections. Previous studies reported that African Americans and females were more likely to work at essential industries or frontline with lower paid which attributed to the differential risk with respect to COVID-19 (35, 36). The COVID-19 pandemic has shown us that public health authorities need to do more preparation in the future to reduce health disparities by providing valuable resources earlier to vulnerable populations for the next pandemic.

This study has several strengths. LTR is one of four central cancer registries that continue collecting statewide population-based PCL cases and we have comprehensive data over time for this special population. The state COVID-19 data that we used to measure the disparities in COVID-19 infection among PCL women included more than 2 years of COVID data collection. Most of the population-based studies that assessed racial/ethnic disparities in COVID-19 infections in the U.S. were based on the proportion of specific racial/ethnic population at county or zip code level (9, 10, 12, 13), except for a study that used electronic health records across 50 states in the U.S. (7). In this study, we collected individual race/ethnicity data directly from medical records and enhanced completeness with several external sources including Hospital Inpatient Discharge Data (HIDD), Department of Motor Vehicle (DMV), Louisiana Immunization Network (LINKS) Central Registry, and LexisNexis Accurint®. Lastly, we used a composite index of census tract-level SES as a proxy for individual SES, which tends to distinguish more homogenous populations with regard to socioeconomic characteristics than county-level or zip code-level (32).

Our study is subject to a few limitations. The Louisiana statewide COVID-19 database might not capture all infected residents because early in the pandemic COVID-19 tests were limited for persons who had more severe symptoms, and later in the pandemic those who tested positive with home rapid test kits were not reported if not confirmed with a positive PCR or antigen test. Furthermore, we did not consider patients’ underlying health conditions in the adjusted model, due to lack of such information among PCL women. Studies showed that certain chronic health conditions, such as diabetes, hypertension, and obesity were associated with increasing COVID-19 infection (11, 14). However, Escobar et al. demonstrated that racial disparity in COVID-19 infection still existed even after controlling for comorbid conditions (11). The census tract-level SES and urban-rural status are based on the address at the time of PCL diagnosis not at COVID-19 testing positive, because we do not collect current residential address for those PCL women without COVID-19 infection. Even though we received census tract code from the LDH for COVID-19 data, over 12% of PCL women with positive COVID-19 had missing census tract. Without a full address for each individual we were not able to identify which census tract was geocoded based on PO Box addresses or ZIP code centroid for COVID-19 infected women which, further, need to be excluded from the data analysis or conduct imputation for missing data. Additionally, PCL women that were not matched with the Louisiana COVID-19 positive database were assumed to be COVID-19 test negative, this could introduce a collider bias or selection bias (37, 38), because PCL women who tested negative may differ from those not receiving a test based on health condition, type of work, and social determinant of health (38). Another limitation is that the individual social behavior, contact pattern, and regional infection mitigation measures were not collected for this study. Although COVID-19 is a transmitted from person to person via respiratory droplets, more analysis needs to be done to quantify the effects of mask wearing and social distancing to reduce infection risk in working-age women with PCL.

## Conclusions

The current study highlights the substantial variation in racial/ethnic and SES inequalities in COVID-19 infections among working-age PCL women, especially for women residing outside of the Greater New Orleans area. The findings of this study reveal that NHB PCL women and women with low SES disproportionately experienced a significant increased risk of COVID-19 infections. Working women with an underlying PCL condition should be strongly encouraged to adhere to all preventative public health measures, such as being fully vaccinated, mask wearing, and maintaining social distance in light of their pre-cancerous diagnosis. Policy makers should identify targeted areas with high COVID-19 infection rates and/or low vaccination rates and provide education, facial masks, and easier access to vaccination to help reduce these disparities in SARS-CoV-2/COVID-19 exposure and infection in Louisiana. It is critical that healthcare policy decision makers continue to be aware of existing health disparities surrounding COVID-19 infections in order to implement targeted measures that will reduce this unequal burden among PCL women through public health interventions and resources.

## Data Availability

The original contributions presented in the study are included in the article, further inquiries can be directed to the corresponding author.
